# Empirical Study of Nova Scotia Nurses’ Adoption of Healthcare Information Systems: Implications for Management and Policy-Making

**DOI:** 10.15171/ijhpm.2017.96

**Published:** 2017-08-13

**Authors:** Princely Ifinedo

**Affiliations:** Department of Financial and Information Management, Shannon School of Business, Cape Breton University, Sydney, NSW, Canada.

**Keywords:** Healthcare Information Systems, Nurses, Technology Adoption, User Behavior, Computer Anxiety, Computer Habit, Computer Knowledge

## Abstract

**Background:** This paper used the Theory of Planned Behavior (TPB), which was extended, to investigate nurses’ adoption of healthcare information systems (HIS) in Nova Scotia, Canada.

**Methods:** Data was collected from 197 nurses in a survey and data analysis was carried out using the partial least squares (PLS) technique.

**Results:** In contrast to findings in prior studies that used TPB to investigate clinicians’ adoption of technologies in Canada and elsewhere, this study found no statistical significance for the relationships between attitude and subjective norm in relation to nurses’ intention to use HIS. Rather, facilitating organizational conditions was the only TPB variable that explained sampled nurses’ intention to use HIS at work. In particular, effects of computer habit and computer anxiety among older nurses were signified.

**Conclusion:** To encourage nurses’ adoption of HIS, healthcare administrators need to pay attention to facilitating organization conditions at work. Enhancing computer knowledge or competence is important for acceptance. Information presented in the study can be used by administrators of healthcare facilities in the research location and comparable parts of the world to further improve HIS adoption among nurses. The management of nursing professionals, especially in certain contexts (eg, prevalence of older nursing professionals), can make use of this study’s insights.

## Introduction

Healthcare information systems (HIS) encompass computer hardware and software used for capturing, processing, storing, retrieving, sharing, and presenting data and information for decision-making in healthcare facilities such as clinics, health centers, and hospitals.^1^ Examples of HIS include electronic medical records (EMR), electronic health records (EHR), mobile nursing information systems (MNIS), clinical decision support systems (CDSS), electronic patient records (EPR), and patient care systems (PCS).^2-7^ Healthcare facilities across the world expend large amounts of money to acquire and maintain various types of HIS.^4,6,8-12^

HIS increase the timeliness and accuracy of patient care and enhance organizational efficiency by reducing costs and improving standards in healthcare delivery.^5,6^ Tung et al^6^ while citing previous work commented that “if hospitals do not adapt [HIS], they will be inefficient and lose the trust of their patients.” Favorable use of HIS ultimately impacts patient satisfaction and outcomes.^13,14^

In spite of the benefits of HIS to healthcare, the literature indicates that in some instances nurses, have been reluctant to accept HIS and have at other times underutilized such technologies.^15-19^ Studies have been conducted in Canada^20-24^ and across the world^2,3,15-19,25-27^ to assess nurses’ adoption of HIS and related technologies. Studies in this study’s location that examined nurses’ perceptions are rare but more are beginning to emerge.^7,28,29^ For example, Ifinedo et al^28^ showed that perceived usefulness of HIS and favorable computer habits have positive effects on Nova Scotia nurses’ acceptance of HIS.

It is argued that knowledge accumulation and theory development with respect to nurses’ adoption of HIS stand to benefit from efforts that present insights and observations from diverse contexts. For instance, Poon et al^8^ noted the adoption of HIS varies markedly between clinicians in America and Holland. The foregoing insight suggests that it may be erroneous to accept that perceptions uncovered in prior studies would apply to all contexts, including Nova Scotia, a province of Canada, and this study’s research setting.

In fact, there are particular socio-economic conditions existing in Nova Scotia that need to be highlighted; chief among these are shrinking economy, low levels of technology acceptance, declining populations, and the presence of the oldest nursing professionals in Canada who have a less than favorable attitude toward new healthcare technologies.^28-32^ In fact, prior healthcare studies have implied that socio-economic factors may permit a better understanding of nurses’ acceptance or rejection of new nursing practices and technologies.^26^ Thus, investigating the realities of this study’s research location is a worthwhile exercise.

Nova Scotia implemented a HIS, ie, NSHIS, at an approximate cost of over $55.7 million; the system is comprised of EMR, EHR, CDSS, EPR, PCS, and related technologies.^9^ No prior empirical information exists regarding adoption of the system. Given that Nova Scotia nurses and other healthcare professionals are mandated to use the system at work, it would be reasonable to gain an understanding of salient factors likely to influence adoption of the acquired system. This present study is designed to enlighten in this aspect. Information from this study would help hospital administrators and policy-makers in Nova Scotia and comparable regions to develop strategies and policies that enhance clinicians’ adoption of HIS and similar technologies.

Results reported in studies conducted in more socio-economically endowed provinces of Canada, ie, Quebec and Ontario, that used one of the commonly used psychosocial behavioral theoretical frameworks for examining healthcare professionals’ adoption of technologies, ie, the Theory of Planned Behavior (TPB),^33^ have produced interesting but mixed results. While Leblanc et al^21^ reported that nurses’ intention to adopt HIS in Quebec was influenced by TPB’s three constructs (attitude, subjective norm, and facilitating conditions), Zhang and colleagues’ study^20^ in Ontario revealed that only subjective norm mattered for nurses’ adoption of HIS. Another study in Quebec demonstrated that specific normative pressures of a nurse’s professional group was the only construct from TPB that predicted nurses’ intention to adopt HIS.^23^ A study of healthcare professionals, including nurses, by Gagnon et al^24^ in Quebec used a related theoretical framework, ie, Triandis’ theory of interpersonal behavior (TIB)^34^ that included TPB’s three constructs. They found that facilitating conditions was the only significant predictor of acceptance of a new telemonitoring system.

Research efforts from across the world that included the variables of attitude, subjective norm, and facilitating conditions have also provided mixed results in relation to the pertinence of these constructs in explicating nurses’ adoption of HIS and other technologies.^35,36^ For instance, subjective norm was found to be the most influential factor in terms of safety behaviors displayed by nurses in a study conducted in Iran.^26^ Studies in Kenya^37^ and Turkey^38^ showed that nurses had favorable attitudes toward computerization. Simpson and Kenrick^16^ also reported that nurses’ attitudes toward computerization in clinical practice in a British general hospital were generally positive; however, they found significant differences in relation to nurses’ age and length of service. Chung and colleagues’ study of nurses’ intention to adopt HIS in Taiwan showed that attitude and subjective norm were key determinants.^39^ In Spain, Asua et al^40^ investigated the acceptance of a type of HIS used by healthcare professionals including nurses. They found facilitating conditions to be significantly related to use behavior; subjective norm did not have a positive impact in their research conceptualization. Shoham and Gonen,^41^ who used TPB in their study of nurses’ intention to use computing technologies at work in Israel, reported that facilitating conditions and attitude were linked to nurses’ intention to use such tools. It remains unclear whether findings from other areas in Canada and elsewhere can be generalized to the context of Nova Scotia. Information from Nova Scotia will add to the growing body of knowledge in the area.

The objective of this study is to apply an extended version of Ajzen’s TPB to investigate factors influencing nurses’ adoption of HIS in Nova Scotia, Canada. The research questions posed in the study are presented as follows:

a) *What factors influence Nova Scotia nurses’ use of HI*S?
b) *What relationship exists among the study’s constructs?*

## Methods

### Theoretical Background

Numerous theoretical frameworks have been used to explain the adoption of a new behavior or technology. This study chose TPB from a list of competing theoretical frameworks^6,42-44^ because this particular social cognitive theory has proven to be helpful in explaining nurses’ use of HIS and related technologies.^45^ Importantly, research has shown that TPB explained significant variance in healthcare professionals’ intention to use technologies.^35,44^ In their systematic review, Godin et al^35^ revealed that TPB explained 59% of the variance of healthcare professionals’ intention to adopt various medical practices and systems to underscore its suitability for this study. Several healthcare researchers, including some cited in this paper, have used the theory in comparable studies. It is worth adding that the adoption of HIS, even in mandatory settings, is still susceptible to the behavioral intentions of healthcare professionals.^35,44^

That noted, TPB posits that individual behavior is influenced by attitude, subjective norms, and perceived behavioral control. Attitude refers to an individual’s positive or negative feelings toward engaging in a specified behavior. Subjective norms describe an individual’s perception of what significant others think about a given behavior. Perceived behavioral control refers to an individual’s beliefs regarding the resources needed to facilitate a behavior. In this study, this variable is described in terms of ‘facilitating organizational conditions’; others have used a similar term.^21,22,24^ Intentions refer to the willingness to engage in a behavior.^33^ As actual usage of HIS was not directly measured in the study, nurses’ self-reported usage of HIS is employed in lieu.

Healthcare researchers^36^ have signified the need for focused attention on issues of concern to specific populations of healthcare workers being studied. Heeding this advice, an informal study was conducted to gauge nursing professionals’ views on factors likely to encourage or discourage adoption of HIS among nurses at work in the research location. The informal study solicited the participation of 6 registered nurses (RNs) in the province; one participant being the former head of the provincial RNs’ association. Among other concerns, participants stressed the inability to use ever-changing healthcare technologies effectively due to the age of nurses, less than desirable computer habits, poor computing competencies, and phobia of technologies. It is difficult to posit with certainty whether the issues noted in the informal study are specific only to this study’s location given that such issues have been highlighted in the literature.^46^ Griebel et al noted that computer competencies and anxiety are among the 10 additional factors recommended for consideration alongside established constructs in technology adoption frameworks for healthcare research.^46^

Accordingly, TPB was extended in this study by three factors, ie, computer anxiety, computer habit, and computer knowledge, that were found to be important for nurses’ adoption of new technologies in the research setting. Computer anxiety refers to a state of heightened tension or a feeling of apprehensive expectation toward computers.^29,47^ Habit, as proposed in TIB, refers to behavior that has become automatic often requiring only minimal mental effort to accomplish. Thus, computer habit refers to a computer behavior that has become automatic.^40,48^ Computer knowledge refers to the skill level an individual has with basic computer hardware and software such as internet browsers, word processors, spreadsheets and presentation software.

### Research Model and Hypotheses

**Figure 1:**
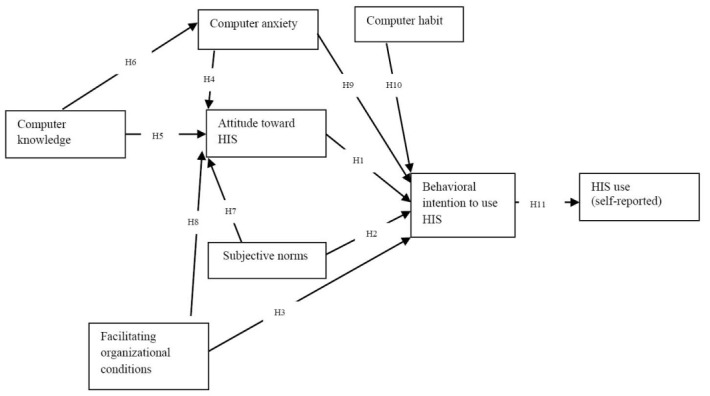
The Research Model.

To understand the phenomenon of nurses’ adoption of HIS in Nova Scotia, it is imperative to base the study on a theory (this has been accomplished). It is also a common practice in quantitative research to draw insights from prior empirical studies and observations in formulating predictions or hypotheses. This study followed such guidelines. The formulated hypotheses and relevant paths or relationships are depicted in Figure 1.

It has been demonstrated that nurses having favorable attitudes toward HIS tend to have willingness to use such systems at work.^3,21,36,41,49^ Nurses tend to use adopted computing systems and technologies at work when their peers use such tools.^20-22,41^ Nurses facilitating organizational conditions matter for the acceptance of HIS and related technologies.^20-22,27^ Namely, higher levels of organizational support and assistance boded well for nurses’ acceptance of technologies.^21,41^ Past studies showed a negative relationship exists between clinicians’ computer anxiety and their attitudes toward technology use.^2,50^ Evidence exists to show that nurses’ computer knowledge and competence enhance positive attitudes toward technology use in work environments.^2,3,7,18^ By the same token, nurses with adequate computer knowledge tend to be less anxious about using technologies for work.^2,50,51^ Thus, the following set of hypotheses are proposed:

H1: Nurses’ attitude toward HIS will have a positive effect on behavioral intention to use HIS
H2: Nurses’ subjective norms will have a positive effect on behavioral intention to use HIS
H3: Nurses’ facilitating organizational conditions will have a positive effect on behavioral intention to use HIS
H4: Nurses’ computer anxiety will have a negative effect on attitude toward HIS
H5: Nurses’ computer knowledge will have a positive effect on attitude toward HIS
H6: Nurses’ computer knowledge will have an inverse relationship with computer anxiety

Studies have shown that peer influence plays a significant role in the behavioral intention of healthcare workers, including nurses, to accept and integrate computing technologies in their work or practice.^21,22,35,41^ Favorable facilitating conditions augured well for nurses’ attitudes toward accepting HIS initiatives and projects in the healthcare industry.^2,3,20,21,27,36^ Kjerulff et al^50^ and Top and Yılmaz^52^ demonstrated that nurses with little or no fear of computing technologies tend to be more willing to accept healthcare tools. Similarly, nurses with well-established computer habits are more accepting of HIS and related technologies in work settings.^53^ Broadly, the relationship between intention and usage behavior has been shown to be consistently strong across contexts.^54^ In the same vein, healthcare researchers have confirmed the strength of the relationship as well.^7,35,41^ Thus, the following set of hypotheses are proposed:

- H7: Nurses’ subjective norms will have a positive effect on attitude toward HIS
- H8: Nurses’ facilitating organizational conditions will have a positive effect on attitude toward HIS
- H9: Nurses’ computer anxiety will have a negative effect on behavioral intention to use HIS
- H10: Nurses’ computer habit will have a positive effect on behavioral intention to use HIS
- H11: Nurses’ behavioral intention to use will have a positive effect on HIS self-reported HIS use

### Population and Study Design

This study used a cross-sectional survey to collect data. A list of 500 names and postal addresses of members of the College of Registered Nurses of Nova Scotia, Canada, (http://www.crnns.ca) was procured. Each person on the list was contacted regarding participating in the survey. Participants were asked to provide views reflecting their use of HIS in their organizations. The definition of HIS with examples was provided to participants. Several participants indicated familiarity with systems such as EMR, HER, CDSS, EPR, and PCS. The developed questionnaire was pretested by 10 university staff and professors and 10 students. 197 usable responses were obtained from the main study to represent a 40% response rate.

### Sample and Instrument

More than 70% of participants had university education and 96% were females, which is an indication of the characteristics of RNs in Canada and elsewhere.^18,55^ The sample included different age categories; however, approximately 75% of the respondents were aged 41 years and above. This information is consistent with data from a report, which indicated that Nova Scotia RNs are the oldest in Canada.^31^ The majority of participants work in public healthcare facilities where HIS are deployed. Most Canadian RNs are government employees.^55^ On average, participants have worked 14.5 years with their current employers (standard deviation [SD] = 10.8). Table 1 shows the rest of the participants’ demographic information.

**Table 1:** Demographic Profile of the Sample (n =197)

**Variable**	**Content**	**Count**	**%**
Gender	Female	189	95.9
	Male	8	4.1
Age	21-30 years	20	10.2
	31-40 years	30	15.2
	41-50 years	52	26.4
	51-60 years	78	39.6
	60 years and above	17	8.6
Education	Diploma	77	39.1
	Bachelor’s	91	46.2
	Master’s	26	13.2
	Doctorate	1	0.5
	Others	2	1.0
Work-related hierarchy	Highest end of career	108	54.8
	Mid-level position	69	35.0
	Just starting out	18	9.1
	Missing	2	1.0
Work location	Urban	112	56.9
	Rural	52	26.4
	Mixed	33	16.8
Work sector	Public	175	88.8
	Private	18	9.1
	Other	4	2.0

Consistent with the survey method, this study used measuring scales validated in prior studies. Items used to operationalize organizational facilitating conditions (FAC), behavioral intention to use HIS (BEH), subjective norm (SUB), and self-reported HIS use (IUS) were adapted from Venkatesh at al.^42^ Measures for computer anxiety (ANX) were adapted from Compeau et al.^56^ Items used to represent computer habit (HAB) were adapted from Limayem and Cheung.^48^ Measures used to capture attitude toward HIS (ATT) were adapted from Davis.^43^ Measurement items were anchored on a 7-point Likert scale ranging from “strongly disagree” (1) to “strongly agree” (7) in which participants were asked to indicate an appropriate response.

For the construct of computer knowledge, participants were asked to indicate their level of skill in terms of software applications such as internet browsers, word processors, spreadsheets and presentation software. The scale used to assess their skill levels was anchored a scale that ranged from “No skill at all” (0) to “Very competent in using it” (6). A full list of measuring items for the constructs and their descriptive statistics is provided in Table 2.

**Table 2:** The Questionnaire’s Items, Their Descriptive Statistics, and Item Loadings

**Construct**	**Measurement Item**	**Mean**	**SD**	**Item Loading**
Computer anxiety (AXT) (Mean = 2.400, SD = 1.670)	I feel apprehensive about using computers	2.827	1.844	0.833
	It scares me to think that I could lose vital information using computers by hitting the wrong key	3.066	1.959	0.787
	I have a fear of computers	2.188	1.535	0.883
	I hesitate to use computers for fear of making mistakes that I cannot correct	2.325	1.695	0.94
	In general, computers are intimidating to me	2.437	1.733	0.944
	I am nervous anytime I find myself sitting behind computers	2.076	1.501	0.913
	Computers make feel uneasy	1.883	1.422	0.807
Computer habit (HAB) (Mean = 4.267, SD = 1.728)	I use computers as a matter of habit	5.096	1.774	0.921
	Using computers has become automatic to me	5.279	1.740	0.953
	Using computers come natural to me	4.777	1.764	0.891
	Using computers has become a habit for me	4.995	1.786	0.946
Facilitating organizational conditions (FAC) (Mean = 5.201, SD = 1.723)	Top management believes that the use of HIS provides significant benefits to the organization	5.629	1.374	0.708
	I receive necessary assistance from my organization that helps me to use HIS	4.812	1.863	0.916
	I have access to resources that would enable me use HIS	5.137	1.746	0.863
	A specific person (or group) is available for assistance with difficulties arising from HIS use	5.406	1.671	0.827
	My organization has helped me in using HIS at work	4.949	1.876	0.896
	In general, my organization has supported the use HIS	5.274	1.809	0.892
Subjective norms (SUB) (Mean = 4.74, SD = 1.574)	People who influence me think that I should use HIS	4.741	1.587	0.947
	People who are important to me think that I should use HIS	4.721	1.600	0.905
	My colleagues think that I should use HIS	4.761	1.535	0.88
Attitude toward HIS (ATT) (Mean = 5.442, SD = 1.383)	Using HIS is a good idea	6.030	1.073	0.776
	HIS make work more interesting	5.355	1.507	0.922
	Working with HIS is fun	5.091	1.495	0.936
	In general, I like working with HIS	5.289	1.458	0.934
Behavioral intentions to use HIS (BEH) (Mean = 6.107, SD = 1.299)	I am certain I will use my organization’s HIS in the coming months	6.107	1.338	0.972
	I predict I would use my organization’s HIS in the coming months	6.102	1.317	0.972
	In general, I intend to follow my organization’s directives to use HIS	6.102	1.237	0.884
	It is my plan to use my organization’s HIS in the coming months	6.117	1.302	0.967
HIS use (IUS) (self-reported) (Mean = 5.005, SD = 1.758)	I frequently use HIS to understand a health problem or an illness	5.178	1.560	0.839
	I often use HIS to serve patients	4.421	1.977	0.773
	I frequently use HIS to find information about a health problem	5.305	1.498	0.879
	I very often use HIS to do my job	5.117	1.998	0.764
Computer knowledge (CNK) Skill level on the following software applications:	Word processing (eg, Ms Word)	3.457	2.093	‏-
	Spreadsheet (eg, MS Excel)	1.970	1.871	‏-
	Presentation software (eg, MS PowerPoint )	2.315	2.222	‏-
	Email	4.853	1.307	‏-
	Web surfing with any Internet browser	4.467	1.615	‏-

Abbreviations: SD, standard deviation; HIS, healthcare information systems.

## Results

### Data Analysis

This study used the partial least squares (PLS) technique for data analysis, which other healthcare researchers have used in similar studies.^4,7,27^ PLS is suitable for this study because it places minimal demands on sample size and residual distributions; it allows the use of observed items to represent a construct.^57,58^ This study used WarpPLS 5.0, which was chosen for its ability to handle both formative and reflective constructs in a model.^59^

PLS tests the reliability and validity of measures. Reliability of reflective constructs with values of 0.6 or more are usually considered acceptable.^58^ Reliability indicators – composite reliability and Cronbach alpha – shown in Table 3 are consistently above 0.6. Regarding validity of the constructs, each reflective measurement item is expected to load highly on its latent construct^58^ and standardized item loadings exceeding 0.707 are considered adequate.^58,59^ Itemloadings shown in Table 2 are all above this threshold. Additionally, a study’s constructs are expected to be distinct.^57-60^ To show distinctness (ie, discriminant validity), a minimum value of 0.5 average variance extracted (AVE) is recommended and the square root of AVE should be larger than correlations between that construct and all other constructs in the model.^60^ Table 3 shows that this study’s constructs meet these criteria.

**Table 3:** CRO, CRA, AVEs, and Inter-Construct Correlations

	** CRO**	** CRA**	** AVE**	**ATT**	**SUB**	**FAC**	**BEH**	**IUS**	**AXT**	**HAB**	**CKN**
ATT	0.941	0.915	0.800	**0.894**	0.377	0.28	0.328	0.446	-0.484	0.592	0.271
SUB	0.936	0.897	0.830	0.377	**0.911**	0.358	0.267	0.337	-0.064	0.25	0.035
FAC	0.841	0.924	0.728	0.28	0.358	**0.853**	0.617	0.499	-0.151	0.401	0.141
BEH	0.973	0.963	0.901	0.328	0.267	0.617	**0.949**	0.529	-0.346	0.477	0.205
IUS	0.888	0.831	0.665	0.446	0.337	0.499	0.529	**0.815**	-0.387	0.602	0.251
AXT	0.958	0.948	0.765	-0.484	-0.064	-0.151	-0.346	-0.387	**0.875**	-0.573	-0.463
HAB	0.914	0.881	0.649	0.592	0.25	0.401	0.477	0.602	-0.573	**0.806**	0.392
CNK	NA	NA	NA	0.271	0.035	0.141	0.205	0.251	-0.463	0.392	**NA**

Abbreviations: CRO, composite reliability; CRA, Cronbach alpha; AVE, average valance extracted; NA, not applicable. Note: Off-diagonal elements are correlations among constructs; The bold fonts in the leading diagonals are the square root of AVEs.

**Figure 2:**
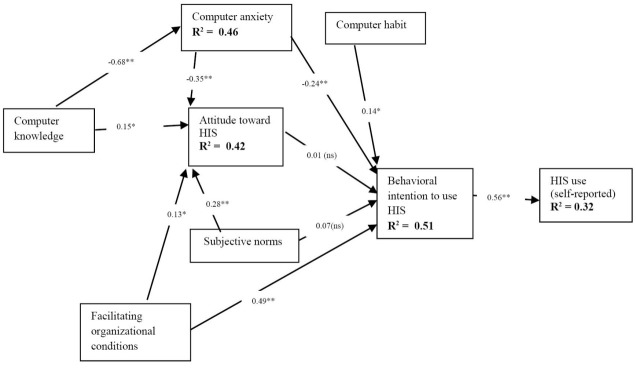
Results of PLS Analysis for the Proposed Research Model. Abbreviations: HIS, healthcare information systems; PLS, partial least squares.

For the formative construct (in bold line in Figure 2), it is recommended that item weights and the presence of multicollinearity be checked.^61^ In assessing multicollinearity among items used to represent computer knowledge, the variance inflation factors (VIF) of items were found to range from 2.112 to 3.217, which are below the threshold cutoff value of 3.33.^61^ In addition, items are expected to have statistical significance.^61^ The items used to represent computer knowledge are significant at the level of *P* < .001 to show that the construct items have adequate psychometric properties.

With reliability, convergent validity, and discriminant validity of the constructs established, the structural model for the hypothesized paths was then tested. The structural model indicates the significance of hypothesized relationships using the path significance (p), beta (β) coefficients, and the coefficient of determination (R^2^), which is the amount of variance explained by the indicators. Table 4 and Figure 2 show the results of PLS analysis and supported hypotheses.

**Table 4:** Results for Statistical Support of the Study’s Hypotheses

**Hypothesis**	**Relationship**	**β**	***P*** ** Value**	**Result**
H1: Nurses’ attitude toward HIS will have a positive effect on behavioral intention to use HIS	ATT (à) BEH	0.01 (ns)	.48	Not supported
H2: Nurses’ subjective norms will have a positive effect on behavioral intention to use HIS	SUB ( à) BEH	0.07 (ns)	.15	Not supported
H3: Nurses’ facilitating organizational conditions will have a positive effect on behavioral intention to use HIS	FAC (à) BEH	0.49**	<.01	Supported
H4: Nurses’ computer anxiety will have a negative effect on attitude toward HIS	AXT ( - à) ATT	-0.35**	<.01	Supported
H5: Nurses’ computer knowledge will have a positive effect on attitude toward HIS	CKN (à) ATT	0.15*	.02	Supported
H6: Nurses’ computer knowledge will an inverse relationship with computer anxiety	CKN ( - à) AXT	-0.68**	<.01	Supported
H7: Nurses’ subjective norms will have a positive effect on attitude toward HIS	SUB ( à) ATT	0.28**	<.01	Supported
H8: Nurses’ facilitating organizational conditions will have a positive effect on attitude toward HIS	FAC (à) ATT	0.13*	<.05	Supported
H9: Nurses’ computer anxiety will have a negative effect on behavioral intention to use HIS	AXT ( - à) BEH	-0.24**	<.01	Supported
H10: Nurses’ computer habit will have a positive effect on behavioral intention to use HIS	HAB (à) BEH	0.14*	<.05	Supported
H11: Nurses’ behavioral intention to use will have a positive effect on HIS self-reported HIS use	BEH (à) IUS	0.56**	<.01	Supported

Abbreviation: HIS, healthcare information systems. Note: Path significance: * *P* < .05; ** *P* < .01, ns = not significant.

Computer knowledge alone explained 46% of the variance in attitude toward HIS. Computer knowledge, subjective norm, computer anxiety, and organizational facilitating conditions explained 42% of the variance in attitude toward HIS. Computer anxiety, attitude toward HIS, computer habit, subjective norm, and organizational facilitating conditions explained 51% of the variance in behavioral intention to use HIS. Behavioral intention to use HIS alone accounted for 32% of the variation in nurses’ self-reported HIS use. Falk and Miller^62^ recommended a minimum value of 0.10 (10%) for R^2^; results in this study are above this threshold. Overall, the results indicate the proposed research model is relevant for theory development.^57,58,62^

## Discussion

This paper describes a study that used TPB, complemented by computer anxiety and computer habit, to investigate nurses’ adoption of HIS in Nova Scotia. Analysis of the collected data showed that 9 out of 11 formulated hypotheses were supported; two were unsupported. The unsupported predictions will be discussed first.

Sampled nurses’ attitudes toward HIS did not positively influence their behavioral intention to use HIS at work. Plausible explanations for this result might be related to extraneous factors in the research setting. The study’s sample was comprised of nurses who were mainly aged 40 years and above, it is likely their attitudes toward implemented technologies may not be as favorable compared to the views of younger colleagues who may be more receptive to technological innovations.^16,38^ In fact, past studies^4,18^ found statistically significant relationships between nurses’ age, attitude toward computers, and intention to use HIS. Participants in the study, especially older nurses, did not seem to have positive attitude toward computerization and innovative medical technologies such as HIS; this might have negatively impacted the result in this aspect. Some feedback representative of older respondents’ comments is provided:

“*I feel that using computers has placed the nurse behind the desk doing computer charting. I feel more time could be spent with patients and giving them support. I think the [HIS] that this province selected is very poor. Some nurses who have returned from [working] in the US tell us of better technologies being used there. Perhaps due to my age and exposure to computers at the later part of my nursing career, I have negative opinions of computers [and of our HIS at work]*” (Staff nurse, aged between 51 and 60 years).

*“In my position I use computer frequently. For many of my colleagues they admit to fear of computer use. The organization supports use of [HIS], but does not invest in educating staff. I feel there is a desperate need to move forward in giving staff the tools they need to best perform at work. More advance computer training and knowledge is urgently needed”* (Nurse/Project Lead, aged between 51 and 60 years).

*“I think [using HIS for work] is a lifestyle for the upcoming youth [ie, younger nurses] and is the way of the future. Too many administrative people take it for granted [that] we are all computer literate when [in fact] we are not*” (Staff nurse, aged between 51 and 60 years).

No meaningful relationship existed between subjective norm and nurses’ intention to use HIS in the study. This result is not in agreement with findings reported in similar Canadian studies and elsewhere.^20,21,23,26,41^ It is, however, consistent with viewpoints indicating that subjective norm is unimportant and is least likely to influence healthcare professionals’, including nurses’ acceptance of HIS and related technologies.^27,35,44^ The variable’s lack of significance in the study also may be related to the fact that most nurses in the sample are about the same age, ie, older, and to a large extent, share the same beliefs about implemented HIS and related technologies in their work environments (see the comments above). These participants may not have the belief that they can influence peers to accept implemented systems at work. The literature suggests that older nursing staff can act as role models for others with regard to technology adoption in hospital settings.^63^ Unfortunately, this may not be possible in the research setting because the sample has a disproportionate number of older nursing professionals. In brief, the result demonstrated that peer influence or social pressure played little or no role in encouraging nurses to accept HIS in Nova Scotia.

The study showed that organizational facilitating conditions encompassing infrastructural, technical, and management support are critically important in stimulating nurses’ willingness to use or accept implemented HIS in work environments. This result mirrors the result reported by others in Canada and elsewhere to affirm that organizational facilitating conditions is the most important factor among TPB’s constructs that influence nurses’ intention to use HIS.^21,24,27,35^ This result indicated that nurses in Nova Scotia who believed their healthcare organizations had high levels of facilitating conditions had corresponding high intention and favorable usage outcomes.

The result supported past findings indicating that nurses with less computer anxiety tend to develop a more favorable attitude toward computerization and implemented HIS and have more willingness to accept and use such technologies in work environments.^27,35,50,52^ Nurses’ computer knowledge was found to have a significant positive effect on attitude toward HIS in the research location. This result is consistent with the viewpoint noting that computer knowledge continues to be viewed as an important factor that can enhance nurses’ acceptance of healthcare technologies.^7,16,18,37,38,49,51,61,64^ A belief that nurses possess adequate computer knowledge or competence may not be true (see comments provided). Recently, Farokhzadian et al^64^ concluded that “Nurses do not have sufficient skills to search best evidence and to use the Internet and online databases for information seeking and retrieval.” Computer anxiety was also shown to be negatively linked to computer knowledge. Notably, nurses who had computer knowledge or skills were less likely to be anxious about using HIS at work. The result supports observations reported in similar studies.^50-52^

The relationship between subjective norm and attitude was confirmed by the data. It is worth noting that “attitude” and “intention to use” are dissimilar constructs. Support and influence from peers or colleagues do impact nurses’ attitude toward implemented HIS and related systems. Others have offered similar insight.^20,23,41^ Nurses in the study showed that where their organizations are able to minimize impediments to HIS utilization or provided necessary assistance to using such systems, attitudes toward the system tend to improve. Previous nursing studies have affirmed the foregoing fact.^2,3,36^ The result showed that nurses’ computer anxiety had a significant negative relationship with intention to use HIS. This result is inconsistent with studies of healthcare professionals, including nurses, that indicated that computer anxiety had no effect on whether a nurse would or would not accept a particular technology.^65^

Nurses’ computer habits are positively associated with HIS usage behaviors. That is, nurses whose computer behaviors have become automatic, over time, tend to be more willing to use implemented HIS at work. This result lends credence to a prior study conducted in Canada^53^; however, it is inconsistent with observations in a Spanish study.^40^ The data confirmed that nurses’ behavioral intention to use HIS strongly influenced self-reported use of the technology. In other words, where the willingness to use implemented HIS was high, favorable levels of acceptance of such systems ensued. Previous studies^7,65^ provided support for the existence of a strong, positive relationship between nurses’ behavioral intention to use information technologies and use outcomes.

### Contribution and Implications for Research

This present study contributes to research literature by demonstrating that TPB is a good theoretical model for predicting and explaining nurses’ adoption of HIS. The extended TPB has more predictive power compared to the original TPB, which explained 39% and 27% of the variances in intention and behavior.^54^ The extended TPB used in this study explained 51% and 32% of variations in intention and usage behavior of HIS, respectively. This study adds to viewpoints indicating that extending or modifying established theoretical frameworks for studying healthcare professionals’ adoption of technologies is a useful exercise.^6,36,46^

While this study’s results, in particular those relating to attitude and subjective norm in TPB agree with previous research findings on clinicians,’ including nurses,’ adoption of HIS and similar technologies in Canada and elsewhere,^20,23,36^ a somewhat interesting result was also noticed. Namely, only one TPB construct was found to be positively related to intention; another study in Canada presented a similar result.^24^ This study argues that extraneous factors (eg, older population of nurses) in the research setting might have influenced the relationships between attitude and subjective norm, and intention in the model. Admittedly, further research is needed to quash or corroborate the foregoing proposition. What is more important is the fact that healthcare researchers need not downplay influences arising from contextual influences in their research locations.

This study’s focus on Nova Scotia with its unique characteristics produced results that are not in total agreement with the tenets of TPB. In light of the growing body of work that used TPB to investigate nurses’ adoption of healthcare technologies and the sorts of results reported in such studies, it may be worthwhile for future inquires to examine the extent to which socio-economic, and even cultural factors impact findings across contexts.

To provide an answer to the question: what factors influence Nova Scotia nurses’ adoption of HIS, this study revealed that organizational facilitating conditions is the most influential variable that predict Nova Scotia nurses’ intention to use HIS. Unfortunately, attitude and subjective norm did not offer meaningful results. With respect to the question dealing with the nature of the relationships among the study’s constructs, it was shown that computer anxiety (lack thereof), computer knowledge, subjective norm, and organizational facilitating conditions are strongly associated with nurses’ attitudes toward HIS. Computer habit and lack of anxiety about computers enhance intention to use HIS. Overall, the study’s results provide further empirical support for challenges espoused by nurses in the research location. Recall, less than desirable computer habits, poor computing competencies, and phobia of technologies are among the inhibitors that were pin-pointed. Future research in the region and comparable locations with similar demographics can build upon the information provided in this study.

### Implications for Practice and Policy-Making

This study has implications for healthcare practitioners. Given that nurses are considered important actors in providing healthcare services to the population, managers of healthcare professionals in Nova Scotia could strive to provide necessary infrastructural, technical, and managerial support to encourage nurses’ adoption of HIS and similar technologies needed to improve healthcare. Comments received from this study’s participants indicated the availability of necessary support (eg, helpful IT desk, training facilities’, system usage manuals) and encouragement from management as worthy of note.

Externally, nurse educators in Nova Scotia and comparable regions of Canada and the world can also play a role in helping curb computer phobia both for older and younger nurses. For example, older nurses can be given short-term courses in nursing informatics and new nurses can benefit from a well-designed curriculum that emphasizes appropriate computing skills and competence.^5,19^ Healthcare organizations ultimately benefit from such programs. A study noted that nursing professionals who received medical informatics classes during their formal education are more likely to have positive attitudes toward technologies used for healthcare delivery.^38^ Older nurses who receive regular computing trainings are also more accepting of computer use at work.^67,68^

Internally, the provision of in-house educational sessions on the importance of implemented HIS for nursing tasks, initial practical training, and support related to newly acquired HIS can help diminish fears associated with using such technologies. Such training, especially for older nurses, could emphasize that mistakes are a normal part of learning and using a new system.^68^ Assurance of this sort can help diminish any phobias, which in turn improve attitudes toward acquired HIS. Management could provide continuous technology training to all nurses. For example, Kuo et al^66^ commented that “continuous educational programs can be provided for nurses to enhance their information technology literacy, minimizing their…discomfort about information technology.”

Because the opinions of peers may influence attitude, it may be worthwhile to encourage nurses’ involvement with implementation teams when new HIS are being procured.^49^ Other nurses would be able to learn from their colleagues and gain first-hand knowledge of how the new system functions; such measures may help improve attitude toward the system.

Administrators could also explore other motivators that will enhance nurses’ general attitudes toward accepting new technologies. For example, incentives (ie, monetary and otherwise) can be given to nurses who utilize implemented HIS effectively in their tasks. Computer habit matters for nurses’ HIS adoption. It may also be rewarding for healthcare establishments to consider developing game applications (apps) that mimic the features and functions of to-be implemented HIS. Such apps (web-based or handheld) can then be made available to clinicians, including nurses, prior to system acquisition or implementation. The supposition is that habits acquired from such endeavors may become automatized to favorably benefit perceptions and attitudes of users toward the actual system when it is eventually deployed.

## Limitations

The results reported in this study should be interpreted against its limitations. First, there is a noticeable gender bias in this study—96% of the participants are females. Nonetheless, the sample was not significantly different from the population of nurses in Nova Scotia and Canada. Second, the data sample was collected from just one region of Canada: Nova Scotia. It is difficult to claim that the results can be generalized to all socio-economically disadvantaged parts of Canada. Third, the study’s participants provided answers to self-reported usage of HIS; the possibility of social desirability bias exists with such an approach. Logs of actual usage of HIS may offer better insight.

Fourth, this study did not focus on any specific HIS. Factors affecting the use of disparate HIS, such as MNIS, CDSS, and EPR, are likely to be different; this reality might be limiting to this study. Fifth, data for the study was obtained in a cross-sectional survey, which only presents a snapshot of insight; a longitudinal study would offer deeper information. Sixth, as it was not explicitly noted that the same measuring items for TPB constructs were used for this study and others, direct comparisons of results should be done with caution.

## Conclusion

In the 21st century, HIS have become a critical resource to improve healthcare delivery across the world, including Nova Scotia. Moreover, acquiring such healthcare infrastructure is very expensive. As such, it is important for researchers to assess the adoption of such technologies by clinicians. This study was conducted in Nova Scotia where HIS has been implemented, but very scant empirical information exists on clinicians,’ including nurses,’ adoption of the technology. This study contributes to the literature by presenting perspective from this setting. Knowledge accumulation and theory development in the area benefit from the endeavor. Using an established theoretical underpinning, ie, TPB which was extended by relevant factors, the study revealed that computer habit, computer knowledge, lack of computer anxiety, and organizational facilitating conditions are among key factors predicting and explaining nurses’ adoption of HIS in Nova Scotia. Information presented in the study can be used by administrators of healthcare facilities in the research location and comparable parts in Canada and elsewhere to design strategies and policies that further encourage HIS adoption among their nurses and other healthcare professionals. Future studies can build upon this work by adding other relevant constructs such as compatibility and self-efficacy.

## Acknowledgements

This study was funded by the Cape Breton Health Research Centre, Competitive Health Research Grant and Cape Breton University, Canada, Research Policy Grant. Assistance, provided by the following individuals (Judy Bailey, Dr. Odette Griscti, Sheila Profit, Cindy Butler, Yvonne Lejeune of Cape Breton University, Canada), is also acknowledged. The author appreciates the support of the College of Registered Nurses of Nova Scotia, Canada and participating nurses in the study.

## Ethical issues

The researchers applied for approval from the research ethics board and committee of the College of Registered Nurses of Nova Scotia, Canada (http://www.crnns.ca) and the researchers’ institution, Cape Breton University, Sydney, NS, Canada (http://www.crnns.ca).

## Competing interests

Author declares that he has no competing interests.

## Author’s contribution

PI is the single author of the paper.

## Key messages

**Implications for policy makers**

Policy-makers can benefit from the results of our study in the following ways:

- Healthcare information systems (HIS) are expensive. Evidence shows that administrators around the world spend millions of dollars to acquire such tools.
- The benefits of HIS will probably not be fully realized if healthcare professionals, such as nurses who are expected to use such tools to serve patients, underutilize them. This is a major challenge to healthcare policy-makers and administrators.
- Encouraging clinicians, including nurses, to adopt HIS and similar technologies is deserving of attention.
- Empirical studies of nurses’ adoption of HIS has the potential to influence policy-making and the management of nurses who use such technologies at work.
- Facilitating organizational conditions where nurses work positively affect their intention to use implemented HIS, so do their computer habits and anxiety (lack thereof).
- Computer knowledge or skills is an important antecedent to acceptance of HIS.

**Implications for public**

In recent years, healthcare researchers across the world have employed antecedent factors such as attitude, subjective norm, and facilitating organizational conditions (perceived behavioral control) to explore nurses’ intention to use implemented healthcare systems and applications. In this study, we used similar factors and others (ie, computer anxiety and computer habit) to examine factors influencing nurses’ intention to use healthcare information systems (HIS) in Nova Scotia, Canada. The study demonstrated that facilitating organizational conditions, computer anxiety, and computer habit meaningfully explained nurses’ intention to use HIS in the research setting. Additionally, computer knowledge mattered for nurses’ acceptance of HIS. The results offer an opportunity for policy-makers and managers of healthcare facilities in the research location to develop appropriate strategies and policies that enhance nurses’ adoption of HIS and similar technologies.
